# Shift Work and Prostate Cancer: An Updated Systematic Review and Meta-Analysis

**DOI:** 10.3390/ijerph17041345

**Published:** 2020-02-19

**Authors:** Mario Rivera-Izquierdo, Virginia Martínez-Ruiz, Elena Mercedes Castillo-Ruiz, Miriam Manzaneda-Navío, Beatriz Pérez-Gómez, José Juan Jiménez-Moleón

**Affiliations:** 1 Department of Preventive Medicine and Public Health, School of Medicine, University of Granada, Avenida de la Investigación 11, Edificio A, 8ª planta, 18016 Granada, Spain; mario.rivera.sspa@juntadeandalucia.es (M.R.-I.); virmruiz@ugr.es (V.M.-R.); ecarui@correo.ugr.es (E.M.C.-R.); miriammanzaneda@correo.ugr.es (M.M.-N.); 2 Service of Preventive Medicine and Public Health, Hospital Clínico San Cecilio, 18016 Granada, Spain; 3 Centros de Investigación Biomédica en Red de Epidemiología y Salud Pública (CIBERESP), 28029 Madrid, Spain; bperez@isciii.es; 4 Instituto de Investigación Biosanitaria de Granada (ibs.GRANADA), 18014 Granada, Spain; 5 Department of Epidemiology of Chronic Diseases, National Centre for Epidemiology, Carlos III Institute of Health, 28029 Madrid, Spain

**Keywords:** occupational health, night work, rotating shift work, circadian disruption, heterogeneity analysis, prostate cancer

## Abstract

The International Agency of Research in Cancer (IARC) has recently confirmed shift work as a type 2A carcinogen. The results presented in published epidemiological studies regarding prostate cancer are inconsistent and the association remains controversial. The aims of this study were: (a) to investigate the possible association between shift work and prostate cancer incidence, identifying possible sources of heterogeneity; and (b) to analyze the potential effect of publication bias. A search for cohort and case-control studies published from January 1980 to November 2019 was conducted. The quality of the articles was assessed using the Newcastle–Ottawa Scale. Pooled OR were calculated using random-effects models. Heterogeneity was evaluated using Cochran’s Q test and data were stratified by potential sources of heterogeneity. Publication bias was analyzed. Eighteen studies were included. No association was found between rotating/night-shift work and prostate cancer, pooled OR 1.07 (95%CI 0.99 to 1.15), I^2^ = 45.7%, *p* = 0.016. Heterogeneity was eliminated when only cohort studies (pooled OR 1.03; 95%CI 0.96 to 1.10; I^2^ = 18.9%, *p* = 0.264) or high-quality studies (pooled OR 0.99; 95%CI 0.89 to 1.08; I^2^ = 0.0%, *p* = 0.571) were considered. A publication bias was detected. An association between shift work and prostate cancer cannot be confirmed with the available current data. Future analytical studies assessing more objective homogeneous exposure variables still seem necessary.

## 1. Introduction

Prostate cancer is one of the most prevalent cancers worldwide, with the second highest incidence rate of cancer, and occupies the sixth position in death rates in males. According to the Global Cancer Observatory (WHO), it is estimated that 1,276,106 new cases were diagnosed around the world in 2018 [1]. As prostate cancer incidence continues to grow, 1,392,797 new cases are expected in 2020 [2].

Despite the high frequency of prostate cancer, little is still known about its etiology. Currently, the list of agents classified by the International Agency of Research in Cancer (IARC) as human carcinogens does not include any exposure with sufficient evidence for prostate cancer [3]. The only associations with this disease that have been consistently proven are genetic susceptibility [4], family history of prostate cancer, advanced age, and black race [5,6,7] all of which are non-modifiable characteristics. Even though several modifiable factors or exposures have been studied as contributors to the origin of prostate cancer, there is not enough evidence to confirm their association with this neoplasm. These factors include (although are not limited to) a fat-rich diet, obesity, physical inactivity, some chronic diseases such as diabetes, and also circadian rhythm disruption [8,9,10,11,12]. Regarding this last exposure, based on human cohort studies and animal experimental studies, in 2007 the IARC classified shift work involving circadian disruption as probably carcinogenic to humans (Group 2A) [13]. As shift work is an occupational factor, there may be some synergic actions with other possible related exposures. Several occupational risk factors have been suspected for prostate cancer, as reviewed recently [14]. However, among several possible occupational carcinogenic agents identified in IARC monographs, no agents for prostate cancer have been detected yet with sufficient evidence [15]. This occupational risk is very relevant in terms of public health, as shift workers represent 18% of all European workers, increasing up to 35% in some countries (e.g., Croatia) [16,17]. These considerations have been reaffirmed in 2019 in the recent reevaluation by an IARC Working Group, reconsidering shift work as Group 2A for prostate cancer with the recent available evidence [18]. This means, as commented by the authors, that there is limited evidence in humans and sufficient evidence in experimental animals [18].

In this sense, the possible involvement of shift work in prostate cancer etiopathogenesis is a plausible hypothesis. Biological clock disruption has already been associated with several cancer types [19,20,21], especially with breast cancer, and there are common mechanistic pathways that could specifically explain its relationship with proliferation of prostate cells; such as an increase in nocturnal light exposure resulting in lower levels of melatonin, which is considered a protective factor for cancer due to its antioxidant effect [22,23]. Other authors have reported that some regulatory components of the circadian rhythm may play a role in DNA damage detection and repair, as well as in the regulation of c-MYC and p53 [24].

From an epidemiological perspective, a number of observational studies have tried to evaluate the association between shift work and prostate cancer, although their results do not always point in the same direction [25,26]. This has led to the conduction of several reviews and meta-analyses on this topic, albeit again with inconsistent results [27,28,29,30,31,32,33]. A detailed analysis reveals that they have significant methodological weaknesses, such as high unexplained heterogeneity [28], partial inclusion of the evidence due to restrictions based on study design [27] or type of exposure (i.e., restricted to permanent night jobs), or the absence of consideration of potential publication bias [32]. The weaknesses and strengths of these reviews are summarized in online Supplementary Table S1. Briefly, some reviews did not include different designs but cohort studies [27], however, it has been suggested that well designed case-control studies may provide more evidence than cohort studies with poor control of biases [34]. Other studies did not consider rotating work as exposure variable [29,30,31]. The consideration of only fixed-night work as exposure explains the lower number of studies included in these reviews, although the IARC considered rotating work among circadian cycle disruptors [13]. In addition, the recent publication of high-quality studies in the last years [35,36,37,38,39,40], not included in prior reviews, gives added justification to the performance of an updated systematic review and meta-analysis. Specially, the most recently published work [39], represents one of the largest and most informative case-control studies to date with detailed exposure information.

In recent years an increase in interest in this topic can be observed, especially from 2007 onwards when the IARC, for the first time, cataloged shift work as a probable human carcinogen. As the IARC is a component agency of the World Health Organization (WHO), its monographs on cancer risks are widely recognized. The current classification of certain types of shift work as “probably carcinogenic to humans” places them in the same risk class as, e.g., ultraviolet radiation, benzo(a)pyrene, and acrylamide [41]. In the case that there was evidence of a relationship between rotating shift work or night shift work and prostate cancer or other cancers, considering them as occupational diseases could be raised, considering that the WHO defines occupational disease as “any disease contracted primarily as a result of an exposure to risk factors arising from work activity”.

The aim of this systematic review is to specifically investigate the possible association between shift work exposure, i.e., rotating shift or fixed night work, and prostate cancer incidence with an updated systematic review and meta-analysis, overcoming the limitations of previous systematic reviews through a rigorous approach based on: (a) the identification of the studies analyzing the association between shift work and prostate cancer; (b) the methodological evaluation of these studies; (c) the analysis of potential sources of heterogeneity related to the populations of study, exposure measurements, and characteristics of prostate cancer cases included; and, if possible, (d) the estimation of the risk associated to this exposure.

## 2. Materials and Methods

### 2.1. Study Design and Selection Criteria

A systematic review and meta-analysis was carried out in accordance with the Preferred Reporting Items for Systematic Reviews and Meta-Analyses (PRISMA) statement recommendations (http://www.prisma-statement.org/) [42]. The predefined criteria for study selection were: (a) provision of original data; (b) human studies; (c) cohort or case-control design; (d) “shift work”, “night work”, and “rotating work” as exposure variables, and “diurnal work” as the reference variable; (e) prostate cancer as the outcome variable; (f) reporting of the magnitude of the association in terms of odds ratio (OR) or relative risk (RR) and their 95% confidence intervals (95% CI), or studies indicating sufficient data to calculate these parameters; and (g) English, Spanish, or French language. All these criteria had to be met by the studies to be selected. We searched for articles published before 1 November 2019. When there were several articles reporting data on the same participants, we used the most recent one.

### 2.2. Search Strategy and Study Selection

The search was performed in the Medline (US National Library of Medicine, Bethesda, MD, USA), Web of Science Core Collection (Thomson Reuters) and Scopus (Elsevier) databases, using both MeSH and free terms to capture all relevant publications. The following terms were combined: (“prostate cancer” or “prostate neoplasms” or “prostate tumor” or “prostatic cancer” or “prostatic neoplasms” or “prostatic tumor”) and (“shift work” or “night work” or “rotating work” or “rotate work” or “shiftwork” or “work schedule”) and (“case-control study” or “cohort study”). The second stage was to apply, in the different databases, the following filters: Humans, Year: after 1980, and Languages: English, French, and Spanish. Duplicates were identified with RefWorks software, and titles and abstracts were independently screened by two researchers to identify the relevant articles. Disagreements in the study selection between the two assessors were resolved by the senior epidemiologists of the research team (VM-R and JJJ-M). After this phase, the full text of the remaining reports was read and reviewed, and their references were examined to look for additional studies of interest not previously found (cluster search). Similarly, the references of the previously identified systematic reviews were also assessed to broaden the cluster search.

### 2.3. Data Extraction and Quality Evaluation

A standardized form was used for extracting the relevant data from each study, namely: (a) first author name; (b) study design (i.e., cohort study or case-control study); (c) publication year; (d) country; (e) research team composition (multidisciplinarity and inclusion of epidemiology or statistics experts were assessed); (f) starting year; (g) population size; (h) follow-up time in cohort studies; (i) type of exposure (i.e., shift work, night work, diurnal work); (j) number of cases and controls in case-control studies and number of exposed and non-exposed participants in cohort studies; (k) association measure: RR/OR and 95% CI; and (l) main confounding variables considered in the study (age, ethnic group, familiar background), and secondary factors (body mass index, smoking, socioeconomic status, etc.).

The Newcastle–Ottawa Scale (NOS) proposed by Wells et al. [43] was used to evaluate the quality of the studies included in the review. Its items can be consulted on the website (http://www.ohri.ca/programs/clinical_epidemiology/oxford.asp). The NOS is adapted to the type of design and allows for the measuring the validity of results depending on the presence of selection, reporting, and confounding biases. According to the NOS score, the selected studies were divided into high quality (8–9 stars), medium quality (6–7 stars) and low quality (5 or less). The scale was applied by two different researchers. Online Supplementary Table S2 presents the agreement between evaluators per item. A 100% unweighted Kappa index (k) was found for 6 items, 2 showed substantial agreement (k: 61–80%) and 5 moderate agreement (k: 41–60%) [44]. Disagreements in the quality assessment between the two assessors were resolved by the senior epidemiologists of the research team (VM-R and JJJ-M).

### 2.4. Data Synthesis and Analysis

The RR, OR and 95 CI% were extracted or, if needed, calculated for each study. All the estimates considered in the meta-analysis were those that were adjusted by the confounders listed in Table 1. To obtain a summary parameter, a random-effects model was applied and weighting by the variability of the studies included. I^2^ estimator and heterogeneity Cochran Q test were used, establishing a p-value of 0.20 to define absence of homogeneity among the results included in the analysis. Heterogeneity was considered as low for I^2^ values between 25% and 50%, moderate for 50–75% and high for >75% [45]. We explored possible sources of heterogeneity by stratifying studies according to several potential candidate variables (subset analyses), namely, quality of the studies according to NOS, period of publication, geographical location and type of exposure (rotating shift work, night work, or both). A random-effects model was also applied for these subanalyses [46]. In addition, publication bias was analyzed using a funnel plot, and the Egger test was applied to confirm if there was any small study effect [47]. The trim-and-fill method was used to estimate the number of unpublished studies and to fill the gaps responsible for the funnel plot asymmetry. For this purpose, input of symmetric variables was done. All statistical analyses were performed using Stata V.15 statistical software (Stata Corp, College Station, TX, USA).

## 3. Results

### 3.1. Study Selection

**Figure 1:**
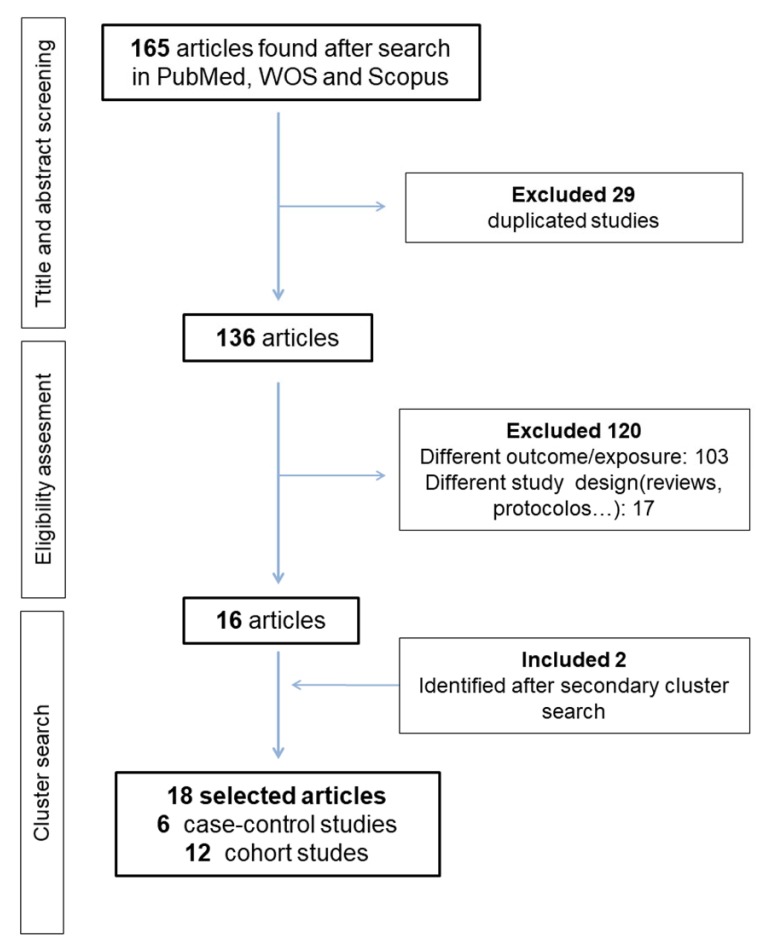
Flow diagram of article selection.

Figure 1 summarizes the selection process of the articles. Our search strategy returned 165 records. After exclusion of duplicates (29 papers) and screening of the title and abstract, 16 studies remained for full text evaluation. All these studies fulfilled the inclusion criteria. A cluster manual search resulted in the location of two additional studies. Thus, a final 18 published studies were included in this review: 12 cohort studies and six case-control studies [25,38,39,40,48,49]. Among cohort studies, 10 collected exposure at recruitment [35,36,37,50,51,52,53,54,55,56] and two included retrospective information collection [24,52]. As two manuscripts provided data from the same cohort [53,54] we only included the most recent one in the meta-analysis [54]. Thus, a total of 17 studies were included in the meta-analysis and 18 studies were included in the systematic review.

The main characteristics of the selected studies are summarized in Table 1. Six out of the 18 reports were conducted in Nordic European countries, in large occupational cohorts linked to national population-based cancer registries. According to the publication year, the earliest study appeared in 2002, but 50% of the articles were published in 2014 or later. 

### 3.2. Methodological Evaluation of the Studies: Identification of Systematic Bias

**Figure 2:**
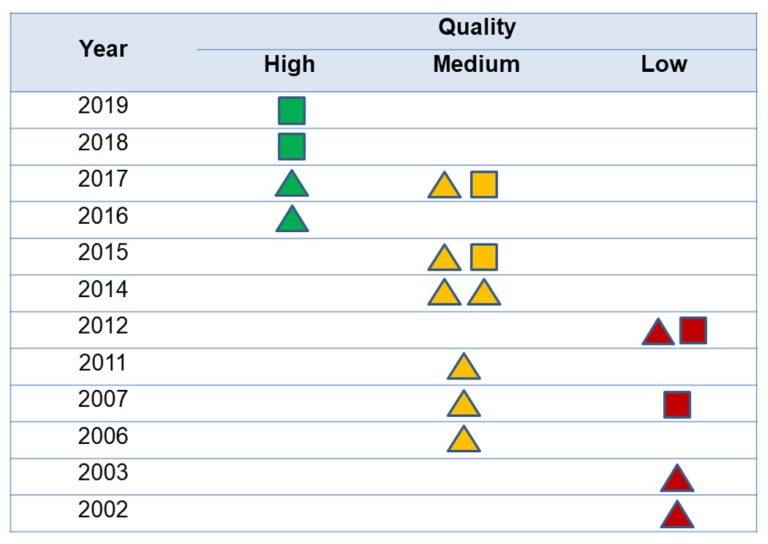
Quality of the studies according to the publication year. The triangles represent cohort studies and the squares, case-control studies. The color represents quality of the studies according to Newcastle-Ottawa scale.

The quality of the studies according to the NOS scale by publication year is shown in Figure 2. Only four studies (22.2%) could be considered as having high quality, and most of the others had medium to low quality. No statistical differences were observed between quality degree and type of design (*p* > 0.05). A trend to quality improvement of studies over the years was observed, with most recent articles showing better quality than previous ones.

### 3.3. Association between Shift Work and Prostate Cancer: Meta-Analysis Results

**Figure 3:**
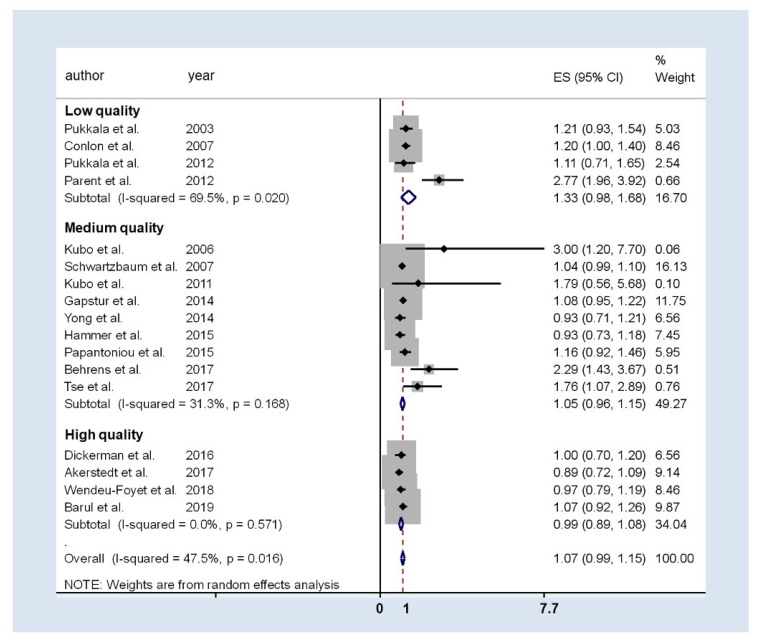
Forest-plot of the association between prostate cancer and rotatory or night shift work. The studies are grouped into three categories depending on the NOS quality detected.

Figure 3 presents the results of the general meta-analysis forest-plot and considers the quality score of the studies. The pooled analysis resulted in an OR of 1.07 (95%CI 0.99 to 1.15), with moderate heterogeneity (I^2^ = 47.5%; *p* = 0.016), but there were differences among quality categories: for high and medium quality studies estimates were similar (OR_high_: 0.99; 95%CI 0.89 to 1.08; OR_medium_: 1.05; 95%CI 0.96 to 1.15), but those with low quality had a higher weighted OR: 1.33 (95%CI 0.98 to 1.68). In addition, in low-quality studies heterogeneity was still high and in medium-quality studies was moderate especially due to two studies: Parent et al. and Behrens et al. [25,36], the studies with lowest sample size and highest OR reported. As these studies may be impacting the meta-analysis results, a sensitivity leave-one-out analysis was done. No differences were detected in the final estimation and, therefore, these studies were included in the final meta-analysis.

According to the definition of exposure, all the 17 studies included in the present meta-analysis evaluated the relationship between shift work and prostate cancer (seven studies evaluated the relationship between rotating shift work exclusively and prostate cancer; three evaluated the relationship between fixed night shift work and prostate cancer, and only seven evaluated both exposures). Only four studies found a significant positive association, two for night work [25,38] and two for rotating shift work [36,52].

The pooled estimate for rotating shift work showed a 5% increase in prostate cancer risk (weighted OR 1.05; 95%CI 1.00 to 1.10), with no heterogeneity (I^2^ = 0.0%; *p* = 0.460) (Table 2).

For fixed night shift work, the association with prostate cancer was analyzed in three studies, two of them reporting a significant positive association [25,38]. The pooled estimate was higher than the one obtained for shift work, although it did not achieve statistical significance (weighted OR 1.81; 95%CI 0.86 to 2.76), while heterogeneity was high (I^2^ = 81.2%, *p* = 0.005).

The stratification by study design (cohorts and case-controls) showed an excess of risk in case-control studies—which presented medium-high heterogeneity—that was not observed in those with cohort design (19% and 3%, respectively). The OR for the case-control studies was 1.19 (95%CI 0.98 to 1.40) compared to cohort studies, OR 1.03 (95%CI 0.96 to 1.10). 

### 3.4. Publication Bias

**Figure 4:**
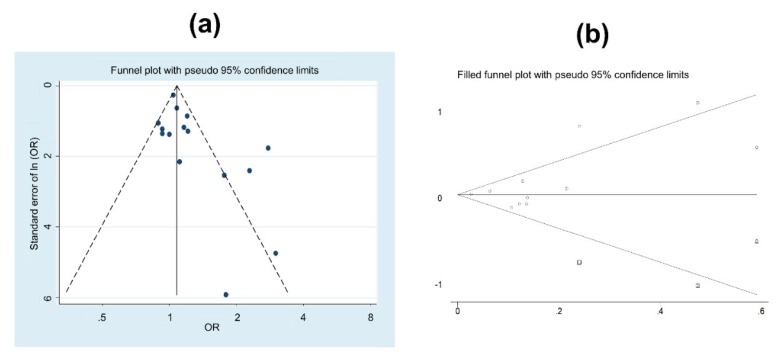
(**a**) Funnel plot of the studies analysing prostate cancer and shift or night work. (**b**) Symmetric funnel plot for the estimation of the studies needed to solve the publication bias.

Figure 4 shows the funnel plot intended to evaluate possible publication bias. The probability of publishing low sample size studies, with higher sample bias, is higher when the estimate indicates an increase in prostate cancer risk: Egger test (*p* = 0.031). A sensitivity analysis demonstrated that exclusion of higher weight studies did not affect the results obtained; except for the study of Akerstedt et al., [35] its exclusion resulted in a weighted OR of 1.11 (95%CI 1.01 to 1.21), but with medium heterogeneity (I^2^ = 49.5%, *p* = 0.015).

A simulation test to estimate the amount of studies needed to overcome the publication bias was performed. The results showed that 3 more studies with protective estimation would be needed to ensure the absence of publication bias (see Figure 4b).

## 4. Discussion

This systematic review summarizes and combines the available information on the association of prostate cancer and rotating shift or night work. Our results do not support the hypothesis that these work schedules are associated with a higher risk of prostate cancer. However, summary measurements must be considered with caution, due to the high heterogeneity found, the high proportion of studies with low-medium methodological quality, and the possibility of publication bias. As we have previously mentioned, other authors have also tried to summarize available evidence [27,28,29,30,31,32,33], as shown in online Supplementary Table S1.

Briefly, a positive association was reported by Gan et al. regarding shift work (RR = 1.23, 95%CI 1.08–1.41) [28], Rao et al. regarding fixed night-shift work (RR = 1.24, 95%CI 1.05–1.46) [29], and Liu et al. regarding fixed night-shift work (OR = 1.26, 95%CI 1.05–1.52) [30]. Mancio et al. [31] reported a positive association for rotating shift work (RR = 1.06, 95%CI 1.01–1.12), but no significant association was shown for fixed night-shift work (RR = 1.01, 95%CI 0.81–1.26). Coinciding with our results, an inconclusive association was reported by Du et al. (RR = 1.08, 95%CI 0.99–1.17) [27], and the systematic reviews conducted by Wendeu-Foyet et al. [32] and Salamanca-Fernández et al. [33].

However, the systematic reviews and meta-analysis published before 2017 did not include the most recent studies, which are precisely the only ones that showed high quality according to the NOS [35,36,37,38,39,40]. In addition, they present unsolved problems that justify the realization of the present review. Wendeu-Foyet et al. [32] and Salamanca-Fernández et al. [33] only developed a systematic review, without including a meta-analysis with the corresponding heterogeneity analysis. The meta-analyses by Rao et al., [29], and Liu et al. [30] only considered fixed night-shift work as the exposure variable, without considering rotating shift work. This explains the lower number of studies they included. Gan et al. [28] also included a lower number of studies and did not observe publication bias. This last systematic review found an increased risk in prostate cancer in personnel working rotating shifts. However, they reported a very high unexplained heterogeneity (I^2^ = 82.7%), and its possible causes were not addressed or resolved. Du et al., [27] in a systematic review published in 2017, included only cohort studies discarding other type of valid designs to address this issue and did not find any association between shift work and prostate cancer when the quality of the studies was considered. In this review, the authors also acknowledged that publication bias could not be ruled out. Finally, a recent meta-analysis [14] analyzed several occupational risk factors for prostate cancer, and shift work showed a significant association (RR = 1.25, 95%CI 1.05–1.49), although with high heterogeneity (I^2^ = 78%). Other occupational risk factors were detected, such us pesticides, chromium, and pilots, and occupational physical activity was detected to be a protective factor. These exposures should be considered when analyzing shift work, as occupational factors might present synergic actions in the development of prostate cancer. It is important to note, however, that among several possible occupational carcinogenic agents identified in IARC monographs, no agents for prostate cancer have yet been detected with sufficient evidence [15].

### 4.1. Methodology of the Studies

The present meta-analysis has been conducted following PRISMA publication guidelines, [42] valid since 2009, that ensure an improvement in the quality and transparency of this type of study. We included both cohort and case-control studies. These studies are often placed immediately below experimental studies in the pyramid of scientific evidence and are the best observational epidemiological studies [34] Nevertheless, this situation depends on the validity of the study, as systematic biases decrease the level of evidence.

In this sense, a factor that must be considered is that most of the studies included in the present systematic review were conducted in occupational cohorts. Several of these studies [53,54,55] used external comparison cohorts, which limited comparability between groups, and a healthy worker effect cannot be discarded. In the same way, some cohort studies were limited to specific occupations, namely pilots or cabin staff. These workers usually must pass exhaustive health tests that may facilitate the diagnosis of prostate cancer. That bias would help to find a positive relationship with cancer incidence, as indolent cases are frequently diagnosed if PSA is indiscriminately used as screening test.

Another relevant issue is the assessment of the exposure variable (shift work), as shifts can be very variable in different work activities, possibly also within the same study, with overlapping of the types of shifts, and often involving subjectively reported data in studies. Furthermore, in the studies where the exposure variable was assessed by asking the participants, a recall bias cannot be discarded. Only sometimes the studies are based on registries with objective data regarding the numbers and type of shifts, and the specific length of the shift, with starting and ending time. Besides, the definition of the exposure variable is not homogeneous in all the studies and may be a possible source of heterogeneity. Some authors have considered “shift work” as only night work, other authors have considered only rotating shift work, and others have considered both kinds of work. Three of the studies [53,54,55] working with cabin staff defined the exposure from the number of flight hours. This definition could have hindered the comparison of their results with those of the other studies. We highly recommend considering night shift work and rotating shift work as exposure variables, as the IARC defines both types of work as shift works able to produce circadian disruption. However, the subsequent analysis should be stratified by both types of work, as current evidence has not still demonstrated the independent effect that each work can have on the development of the outcome. We found that exposure assessment of night work was very different between studies, and thus we believe that scientific literature would benefit from data pooling efforts and uniform definitions of night shift work for future studies. In this sense, authors believe that the accurate complete definition of the exposure variable shown in the two most recent published works [39,40] should be standard and emulated for future works in order to homogenize and avoid heterogeneity.

Additionally, the confounding factors considered in each of the studies were different. Regarding the outcome variable, no variability was found, as prostate cancer diagnoses were identified from national registers and histologically confirmed in most of the included studies. However, only four studies collected data on the severity of prostate cancer and stratified the results by degree of severity [38,39,40,49], and the rest of the studies considered all prostate cancer as equal [25,26,27,35,36,48,50,51,52,53,54,55,56,57].

### 4.2. Discussion of the Results

The weighted OR does not show a significant association between rotatory shift or night shift work and prostate cancer. The heterogeneity found, around 50%, does not completely allow consideration of the result obtained as a reliable estimate of the associated risk. Hence the importance of the analysis to identify sources of heterogeneity.

#### Design

Heterogeneity disappeared when working only with cohort studies, but no significant association was found (OR 1.03; 95%CI 0.96 to 1.10). Any increase of risk with a magnitude strength lower than 1.20 could be explained by the presence of systematic biases. Only 25% of cohort studies showed excellent quality of methodology. Two of these studies did not find an association, thus contradicting the third one. Case-control studies found a higher risk association, but heterogeneity was unacceptable, and no signification was detected. Regarding quality, only two case-control studies was classified as excellent (the most recent ones). This pattern has also been reported for similar associations: case-control studies estimated higher risks for the association of shift work and breast cancer than cohort studies [58].

#### Methodological Quality of Studies

Quality of more than 75% of the studies was medium-low. Studies with lower quality showed a higher risk of prostate cancer. This phenomenon has been widely described, as lower sample size studies with positive associations are more likely to be published. In 2005, Ioannidis et al. described this phenomenon as false positive findings, pointing out that “*it can be proven that most claimed research findings are false […] there is a widespread notion that medical research articles should be interpreted based only on p-values*” [59]. As commented on previously, studies with low quality, high number of biases and significant p-values are published but their results must be considered with caution.

#### Exposure Variables

Shift work was associated with prostate cancer, with a risk increase of 7% (95%CI 0.99 to 1.15). This slight association, however, could be explained by any bias. Heterogeneity showed borderline values. I^2^ evidenced that 16% of variability could be explained by other factors differing from random [45]. Regarding night shift work, no significant results were found, and heterogeneity was unacceptable. The different assessment of the exposure variable was the main source of heterogeneity and the main reason why there were so few studies evaluated as high quality according to NOS. To achieve a more homogeneous assessment of the exposure variable in future studies, we recommend using a standardized definition of night shift work, as the one considered by Wendeu-Foyet et al. [40]: workers who performed at least 270 h of night work per year or three nights per month during at least 1 year. However, international research guidelines for defining shift work seem to be necessary to improve and homogenize future shift work research.

#### Stratified Analysis

The association observed seems to depend inversely on the quality of the studies. Thus, a higher magnitude in the association is shown when lower quality studies are analyzed. Concretely, all low-quality published studies showed a positive association between prostate cancer and shift work, but only one reached statistically significant results [25]. In this study, the presence of systematic biases cannot be ruled out, and relevant confounding factors were not controlled. Medium-quality studies reported no conclusive results regarding the association between prostate cancer and shift work or night work. With regard to night shift work specifically, none of the studies showed significant results, and the punctual estimators of the strength of the association were completely inconsistent: Gapstur et al. [50] presented a protective punctual estimation OR 0.72 (95%CI 0.44 to 1.18), while a 2.3 RR (95%CI 0.6 to 9.2) was reported by Kubo et al. in 2006, [52] and Papantoniou et al. in 2015 [49] showed an estimation closer to 1 (OR 1.14; 95%CI 0.94 to 1.37). All high-quality studies, the most recently published, consistently reported no association, with OR measures around 1.

#### Publication Bias

It is evident that publication bias cannot be discarded in this meta-analysis. The analysis of the publication bias highlighted the absence of studies with a low sample size that showed no significant association. Therefore, any interpretation of the results should consider that possible bias. The Egger test showed higher probabilities of publication when the studies identified shift work as a prostate cancer risk [60].

### 4.3. Strengths and limitations of the meta-analysis

In this study, a detailed analysis of the quality of the studies has been performed using the NOS, a tool that has proven to be useful in similar systematic reviews [61,62]. The present meta-analysis included cohort and case-control studies, after a selective search in different databases and subsequent cluster search from the references of selected studies. This searching strategy has not been previously performed or, at least, it has not been explicitly described in the methodology, which might explain why the number of studies included in this work is higher than in previous reviews [26,27,28], as well as the inclusion of the new articles published during the last year. Both the evaluation of quality and data collection was performed by two different researchers, in a blind independent manner. Later, senior researchers evaluated and amended the resulting data. This strategy aimed to ensure a serious and rigorous methodological evaluation, avoiding subjective belief-based evaluations. The conscientious approach of publication bias has not been previously performed, and the heterogeneity analysis highlighted that no definitive risk estimation can be effectively considered.

Regarding limitations, many studies included in the meta-analysis were based on general or job-specific occupational cohorts [26,51,52,53,54,55,56,57]. That could prevent its extrapolation to the general population. In addition, variability in the design of the studies generated non-homogeneous results and the inadequate definition of exposure variable cannot be forgotten. This is a well-known and still unsolved problem of this occupational exposure, and represents the main limitation of the study, as well as the absence of severity assessment of prostate cancer in the studies included. The available information does not allow for the confirmation that rotating shift or night-fixed shift work are casual factors for prostate cancer. Furthermore, as reported for breast cancer, a pooled analysis of case-control studies [63] suggested that cohort studies may have included older participants where the exposures were in the distant past. Therefore, the average age at recruitment and follow-up times would have been an interesting source of heterogeneity to analyze. However, many of the studies included in this meta-analysis did not report these data and thus, it was not possible to analyze the possible effect of both these factors.

One of the weaknesses of our study lies in not having contacted the authors of previous works in order to identify possible unpublished material, as no gray literature was included in this study. Similarly, the search strategy conducted in this study might be very specific and not as sensitive, and only three languages were considered. However, the cluster search of the identified studies and the previous systematic reviews yielded only two more studies and, therefore, the weaknesses in our search are not expected to be high. Having access to a register of observational studies that have been carried out would be ideal, similarly to current clinical trial registers [64], and would overcome publication bias. It is necessary to boost the registration of all observational studies once they start in order to control for possible publication biases. Otherwise, it would be important to start high-quality methodological studies working with population samples, correctly defining the exposure variable over the life-course, controlling confounding factors and making homogeneous uniform measurements of the outcomes in all the groups.

### 4.4. Consequences and Applicability of the Results

Including prostate cancer as occupational disease would imply changes in occupational politics and a potential path to improve the prophylaxis and treatment of this prevalent disease. However, we cannot establish that a real association exists after having done the present meta-analysis of the updated accumulated evidence. The authors hope that the conscientious analysis of the heterogeneity sources, especially regarding exposure assessment, may serve as a tool for a better design of future studies.

## 5. Conclusions

This systematic review summarizes the currently available epidemiological evidence on the association between prostate cancer and rotating shift or night-fixed shift work. After analyzing the studies identified in our search, a significant association between shift work and prostate cancer was not detected. Most of the studies included showed medium or low quality, mainly due to the difficulties on the exposure assessment, and those with lower quality reported higher risk estimations. In addition, publication bias cannot be discarded, as lower size studies are more likely to be published when a positive association is found. Finally, high heterogeneity was found due to possible selection biases, information biases resulting from poor definitions of the exposure variables, and confounding factors. The development of new studies on this issue, with higher quality methodology (as those published in the last two years), and a thorough assessment of work schedules is still needed to reach a definitive conclusion.

## Figures and Tables

**Table 1:** Main characteristics of the selected studies.

Study, Year	Country	Population Analyzed	Sample Size	Exposure Variable/s	Outcome Ascertainment Source	Adjustment Variables	Total Quality Score ^1^
Cohort Studies							
*Pukkala* et al. *2002* ^2^	Nordic countries	Pilots cohort (1943–1997)	10,032	Airline-related shift work	National population-based cancer registries	Age, number of block hours in long haul aircraft, country, estimation radiation dose	5
*Pukkala* et al. *2003*	Nordic countries	Pilots cohort (1943–1997)	10,051	Airline-related shift work	National population-based cancer registries	Age, number of block hours in long haul aircraft, country, estimation radiation dose, altitude	5
*Kubo* et al. *2006*	Japan	Industry workers (1988–1997)	14,052	Night and rotating work	PC recorded by national registries	Age, FHPC, study area surveyed, BMI, smoking, alcohol drinking, job type, PA at work, workplace, perceived stress, EL, MS	7
*Schwartzbaum* et al. *2007*	Sweden	Industry workers (1960–1989)	2,102,126	Shift work	National population-based cancer registry	Age, socioeconomic status, occupational position and country of residence	7
*Kubo* et al. *2011*	Japan	Industry workers (1981–2009)	4995	Shift work	Health insurance records	Age, BMI, alcohol intake, smoking, PA and MS	7
*Pukkala* et al. *2012*	Nordic countries	Flight cabin workers (1947–1997)	1559	Airline-related shift work	National population-based cancer registry	Age, sex, country, time since first employment	5
*Gapstur* et al. *2014*	United States	Volunteers from general population (1982–2010)	294,440	Night and rotating work	Mortality by PC recorded by the National Death Index	Age, race, EL, BMI, smoking history, FHPC, frequent or painful urination	7
*Yong* et al. *2014*	Germany	Chemical industry workers (2000–2009)	27,828	Shift work	Regional population-based cancer registry	Age, smoking status, job level, and employment duration	6
*Hammer* et al. *2015*	Germany	Chemical industry workers (2000–2009)	27,828	Shift work	Regional population-based cancer registry	Age, occupational task, and duration of employment	7
*Dickerman* et al. *2016*	Finland	Twins cohort (1981–2012)	11,370	Night and rotating work	National population-based cancer registry (histologically verified).	Age, EL, BMI, PA, social class, smoking status, alcohol use, snoring, zygosity	8
*Akerstedt* et al. *2017*	Sweden	Twins cohort (1998–2003)	12,322	Night and rotating work	National population-based cancer registry	Age, EL, tobacco use, alcohol use, PA, BMI, have children, coffee use and previous cancer	8
*Behrens* et al. *2017*	Germany	Population from highly industrialized zone (2000–2014)	1757	Night and rotating work	Incident primary PC reported in the survey and subsequently evaluated in patient records.	Smoking status, FHPC, EL, equivalent income	7
Case-control studies							
*Conlon* et al. *2007*	Canada	General population (1995–1998)	2392: 760 cases, 1632 controls	Full-time rotating shift work	PC registry-identified cases	Age and FHPC	5
*Parent* et al. *2012*	Canada	General population (1979–1985)	912: 400 cases, 512 controls	Night shift work	Incident PC and other cancers, pathologically confirmed	Age, ancestry, EL, family income, respondent status, smoking, alcohol, BMI, farming, PA	5
*Papantoniou* et al. *2015*	Spain	Hospital cases. Ambulatory controls, (2008–2013)	2483: 1095 cases, 1388 controls	Night shift work	Incident PC, histologically confirmed	Age, center, EL, FHPC, PA, smoking status, past sun exposure and meat consumption	7
*Tse* et al. *2017*	China	Hospital cohort (2011–2016)	833: 431 cases, 402 controls.	Night shift work	Incident prostate cancer, confirmed by histology	Age, MS, employment, FHPC, consumption of deep-fried food and pickled vegetable, green tea drinking habits, bisphenol A exposure	6
*Wendeu-Foyet* et al. *2018*	France	General population (2012–2013)	1698: 819 cases, 879 controls	Night and rotating work	Incident prostate cancer, confirmed by histology	Age, ethnic origin, FHPC, BMI, EL, PA, sleep duration, Gleason score, chronotype	8
*Barul* et al. *2019*	Canada	General population (2005–2012)	3869: 1904 cases, 1965 controls	Night and rotating work	Incident prostate cancer, confirmed by histology	Age, ethnic origin (ancestry) and EL. Lifestyle and occupational variables were not included according to a Directed Acyclic Graph.	9

^1^ Quality of the studies according to the Newcastle-Ottawa Scale (NOS): high quality (8–9 stars), medium quality (6–7 stars) and low quality (5 or less). ^2^ Data from Pukkala et al., 2002 [53], have not been considered for including the same results as Pukkala et al., 2003 [54]. FHPC, family history of prostate cancer; BMI, body mass index; PC, prostate cancer; MS, marital status; EL, educational level; PA, physical activity.

**Table 2:** Subgroup analyses of the relationship between shift work and prostate cancer.

Subgroup Items	Number ^1^	Pooled Results	Heterogeneity (I^2^), %	*p*-Value of Heterogeneity
Exposure variable				
Rotating shift work exclusively	7	1.05 (1.00–1.10)	0.0	0.460
Night-fixed work exclusively	3	1.81 (0.86–2.76)	81.2	0.005
Both	6	1.01 (0.87–1.16)	46.0	0.099
Study design				
Cohort	11	1.03 (0.96–1.10)	18.9	0.264
Case-control	5	1.28 (0.98–1.58)	68.7	0.007
Study quality				
High	4	0.99 (0.80–1.17)	0.0	0.108
Medium	9	1.06 (0.99–1.12)	31.3	0.331
Low	3	1.52 (0.87–2.18)	69.5	0.008
Region of the study				
Nordic European countries	5	1.03 (0.98–1.08)	0.0	0.431
Central-Southern European countries	5	1.02 (0.85–1.18)	44.5	0.125
Asian countries	3	1.84 (1.01–2.67)	0.0	0.771
United States and Canada	3	1.19 (0.97–1.41)	75.4	0.007

^1^ The study conducted by Pukkala et al. [53] was not included analyses for providing data from the same cohort as Pukkala et al. [54]. Thus, a total of 16 out of 17 studies were included in the stratified analysis.

## Supplementary Materials

The following are available online at https://www.mdpi.com/1660-4601/17/4/1345/s1, Table S1 (recent systematic reviews and meta-analysis published) and Table S2 (items analysed according to the Newcastle–Ottawa Scale. Agreement between evaluators).
