# Supplementary material for: Shift Work and Prostate Cancer: An Updated Systematic Review and Meta-Analysis
Source: Int J Environ Res Public Health. 2020 Feb 19;17(4):1345. doi: 10.3390/ijerph17041345 (PMC7068593; doi:10.3390/ijerph17041345)
Supplement: Supplementary file 1 [file ijerph-17-01345-s001.pdf]

Table S1. Recent systematic reviews and meta-analysis published.

Study	Systematic review	Meta-analysis	Studies included	Types of studies	Timeframe of search	Heterogeneity analysis	Evaluation of PB	Result of PB	Exposure variable
Rao et al. 2015	✓	✓	8	Cohort and C-C	< December 2014	✓	✓	X	Fixed night-shift work
Wendeu-Foyet et al. 2017	✓	X	12	Cohort, C-C, ecological	November 2011 to September 2016	X	X	Not analyzed	Circadian disruption
Du et al. 2017	✓	✓	9	Cohort	< February 2017	✓	✓	✓	Shift-rotating and night work
Gan et al. 2018	✓	✓	15	Cohort and C-C	< September 2017	✓	✓	X	Shift-rotating work
Mancio et al. 2018	✓	✓	9	Cohort and C-C	< November 2016	✓	✓	X	Fixed night-shift work
Liu et al. 2018	✓	✓	9	Cohort and C-C	< May 2018	X	✓	✓	Fixed night-shift work
Salamanca-Fernández et al. 2018	✓	X	8	Cohort and C-C	< October 2017	X	X	Not analyzed	Fixed night-shift work

PB, Publication Bias; C-C, Case-Control.

Table S2. Items analyzed according to the Newcastle–Ottawa Scale (NOS). Agreement between evaluators.

Case-control studies	Number of Categories	Maximum difference	% total agreement	% expected agreement	Kappa index	Association strength [1]
**Selection**						
Is the case definition adequate?	3	0	100.0	62.5	1.0	Perfect
Representativeness of the cases	2	0	100.0	62.5	1.0	Perfect
Selection of Controls	3	1	75.0	50.0	0.5	Moderate
Definition of Controls	2	0	100.0	62.5	1.0	Perfect
**Comparability**						
Comparability of cases and controls on the basis of the design or analysis	2	1	75.0	75.0	0.0	Low
**Exposure**						
Ascertainment of exposure	5	2	50.0	50.0	0.0	Low
Same method of ascertainment for cases and controls	2	0	100.0	50	1.0	Perfect
Non-Response rate (dropouts)	3	2	75.0	43.8	0.6	Moderate
**Cohort studies**						
**Selection**						
Representativeness of the exposed cohort	4	0	100.0	51.3	1.0	Perfect
Selection of the nonexposed cohort	3	0	100.0	62.5	1.0	Perfect
Ascertainment of exposure	4	1	91.7	37.5	0.9	Almost perfect
Demonstration that outcome of interest was not present at start of study	2	1	91.7	91.7	0.0	Low
**Comparability**						
Comparability of cohorts	2	1	83.3	54.2	0.6	Moderate
**Outcome**						
Assessment of outcome	4	1	83.3	61.1	0.6	Moderate
Was follow-up long enough for outcomes to occur	2	1	83.3	61.1	0.6	Moderate
Adequacy of follow up of cohorts	4	3	83.3	35.4	0.7	High

[1] The association strength was considered as perfect if kappa index was 1, almost perfect if kappa index ranged from 0.81 to 0.99, high if kappa index ranged from 0.61 to 0.80, moderate from 0.41 to 0.60 and low if kappa index was 0.40 or less.
